# Solution NMR structure of the TRIM21 B-box2 and identification of residues involved in its interaction with the RING domain

**DOI:** 10.1371/journal.pone.0181551

**Published:** 2017-07-28

**Authors:** Amélie Wallenhammar, Madhanagopal Anandapadamanaban, Alexander Lemak, Claudio Mirabello, Patrik Lundström, Björn Wallner, Maria Sunnerhagen

**Affiliations:** 1 Division of Chemistry, Department of Physics, Chemistry and Biology, Linköping University, Linköping, Sweden; 2 Princess Margaret Cancer Center and Department of Medical Biophysics, University of Toronto, Toronto, Canada; 3 Division of Bioinformatics, Department of Physics, Chemistry and Biology, Linköping University, Linköping, Sweden; University of Liverpool, UNITED KINGDOM

## Abstract

Tripartite motif-containing (TRIM) proteins are defined by the sequential arrangement of RING, B-box and coiled-coil domains (RBCC), where the B-box domain is a unique feature of the TRIM protein family. TRIM21 is an E3 ubiquitin-protein ligase implicated in innate immune signaling by acting as an autoantigen and by modifying interferon regulatory factors. Here we report the three-dimensional solution structure of the TRIM21 B-box2 domain by nuclear magnetic resonance (NMR) spectroscopy. The structure of the B-box2 domain, comprising TRIM21 residues 86–130, consists of a short α-helical segment with an N-terminal short β-strand and two anti-parallel β-strands jointly found the core, and adopts a RING-like fold. This ββαβ core largely defines the overall fold of the TRIM21 B-box2 and the coordination of one Zn^2+^ ion stabilizes the tertiary structure of the protein. Using NMR titration experiments, we have identified an exposed interaction surface, a novel interaction patch where the B-box2 is likely to bind the N-terminal RING domain. Our structure together with comparisons with other TRIM B-box domains jointly reveal how its different surfaces are employed for various modular interactions, and provides extended understanding of how this domain relates to flanking domains in TRIM proteins.

## Introduction

Ubiquitination is a vital post-translation modification for many cellular processes including protein turnover in the cell, cell-cycle control, transcriptional regulation, intracellular signaling and innate immunity [1,2]. Protein ubiquitination is a multi-enzyme process involving the attachment of the small protein ubiquitin to target proteins. The ubiquitin-signaling pathway involves a cascade of enzymes—E1 activating enzyme, E2 conjugating enzyme, and E3 ligase–and results in the attachment of ubiquitin on the substrate or on a growing polyubiquitin chain [3]. In humans, genes encoding these three enzymes comprise 2 E1s, ~40 E2s and ~617 E3s [4]. Based on the conservation of structural domains and the mode of action of ubiquitin transfer, E3 ubiquitin ligases are classified in three different classes. The largest family of E3s comprises the Really Interesting New Gene (RING)-type, which are characterized by a direct transfer of ubiquitin from the E2 conjugating enzyme to the substrate, by simultaneous binding of the ubiquitin-conjugated E2 (E2~Ub) and the substrate [5].

The TRIM (Tripartite Motif) family of proteins constitutes the largest subfamily of RING E3 ligases, and is characterized by the RBCC fold, comprising a RING-finger domain, one or two B-box domains, and a coiled-coil domain (CC) [6,7]. Proteins containing a B-box domain hold various functions, and include transcription factors, ribonucleoproteins and proto-oncoproteins [7,8]. The B-box is a unique, RING-like Zn^2+^-binding entity of the TRIMs that is only found among members of this protein family. The B-box conservation pattern is similar to that of the RING [9] [10], but holds a consensus sequence that also includes histidines. Two types of B-boxes, type1 (B1) and type2 (B2), have different length but share a similar but distinctive pattern of cysteine and histidine residues: C-X_2_-C-X_7-12_-C-X_2_-C-X_4_-C-X_2_-[CH]-X_3-4_-H-X_4-9_-H and C-X_2_-H-X_7-9_-C-X_2_-[CDHE]-X_4_-C-X_2_-C-X_3-6_-H-X_2-4_-[CH] for B1 and B2 respectively; the main difference is in the second putative Zn^2+^ site, where type1 displays a cysteine where as a histidine is found in type2 [11]. TRIM proteins can contain both B1 and B2 arranged in tandem, but when there is only one B-box present it is most often a B2 [6] [7] [12]. TRIM proteins are divided into different subclasses depending on the number and type of B-boxes as well as the identity of the C-terminal substrate-binding domain [6,7]. Structures of several TRIM B-boxes have been determined, including B-box type1 structures of TRIM18/MID1 and TRIM19, and B-box type2 structures of TRIM1/MID2, TRIM5α, TRIM18/MID1, TRIM29, TRIM39, TRIM41, TRIM54, and TRIM63/MuRF1 (S1 Table). All these structures displays a RING-like ββα core fold wherein two zinc atoms are coordinated in a classical RING-like cross-braced topology involving four ligand pairs forming two zinc-binding sites, site I and site II.

While no specific biological function has yet been assigned to the B-box domain, one hypothesis is that the B-box directs quaternary structures of the multimodular TRIM proteins, which could then be affected by the absence or presence of further binding partners. The only complex involving the B-box and another domain is the crystal structure of the TRIM5α B-box-coiled-coil (BCC) region [13], a TRIM protein acting as a restriction factor that blocks retroviral infections [14]. This structure gives a structural glimpse of the role of the extended anti-parallel helices as for RING and B-box effector domains to recruit the components necessary for protein ubiquitination and immune innate signaling. The B-box2 domain of TRIM5α has been shown to be involved in higher-order self-association and was thereby suggested to facilitate substrate ubiquitination [15]. Similarly, the TRIM63 B-box2 was shown to contribute in a Zn^2+^-dependent manner to the overall assembly of the TRIM63 oligomer [16]. There are still no structures of a B-box domain jointly together with its corresponding RING domain, but several structures suggest B-box interdomain contacts in TRIM proteins. The B-box2 of TRIM63/MuRF1 has been shown by crystallography and NMR to self-associate into a dimer, mediated by the helix in the conserved ββα-fold (PDB ID: 3DDT, 2D8U) [16]. The two sequentially arranged B-boxes of TRIM18/MID1 (2JUN) fold towards each other, by means of residues on the turn separating the short β-strands in the conserved ββα fold of B-box2 packing against the outer surface of the β-strands of B-box1 [12]. This quaternary arrangement is reminiscent of RING intermolecular heterodimers as BARD1-BRCA1 and the homodimer of HDM2, hence suggesting a regulatory or modulatory function for B-box2 [12].

TRIM21, also known as Ro52, comprises an N-terminal RING, a type2 B-box, a coiled-coil domain and a C-terminal substrate-binding B30.2 (SPRY) domain. TRIM21 has shown to be a target for autoantibodies in patients with the autoimmune diseases Sjögren’s syndrome and systemic lupus erythematous (SLE) [17]. As an E3 ligase, TRIM21 act as a negative regulator of the innate immune signaling system by ubiquitinating interferon (IFN) regulatory factors (IRFs) such as IRF3, IRF5, IRF7 and IRF8 [18] [19] [20] [21]. TRIM21 B-box2 displays an arrangement of the type Cys-X_2_-Asp-X_13_-His-X_2_-His, similar to that observed for the solution structures of the B-box type2 of TRIM5α, TRIM39 and TRIM41 (S1 Table).

In this work, we present the solution structure of the TRIM21 B-box2 domain, and show how this entity interacts with its corresponding TRIM21 RING domain. By comparing our results with previous structures as well as interaction patterns identified for other TRIM B-boxes, we show how different surfaces of the B-box motif are employed for various modular interactions. The work presented here extends the structural understanding of the role of the B-box domain, and how this domain is related to flanking subunits in TRIM proteins.

## Materials and methods

### Protein expression and purification

The plasmid pET28b containing the TRIM21 B-box_86-130_ including an N-terminal 6×His tag, was transformed into *E*. *coli* strain BL21 Codon plus (Stratagene) and grown overnight at 37°C in LB-medium. M9 base medium supplemented with ^13^C –glucose (3 g/l), ^15^NH_4_Cl (1.5 g/l), kanamycin (50 μg/ml) and chloramphenicol (34 μg/ml), was inoculated with the overnight culture. Expression was induced at an O.D._600_ level of 0.9 with 0.5 mM isopropyl 1-thio-β-D-galactopyranoside (IPTG), and incubated overnight at 18°C. To ensure stable protein during the expression, 10 μM ZnCl_2_ was added after induction. Cells were harvested by centrifugation at 3000 rpm at 4°C for 30 min, resuspended in lysis buffer (50 mM Tris-HCl pH 8, 300 mM NaCl, 20 mM imidazole, 10% glycerol, 5 U/ml DNaseI, cOmplete EDTA free protease inhibitor (Roche), 10 μM ZnCl_2_) and finally sonicated on ice. After centrifugation (14 000 rpm, 4°C) for 60 min, purification was performed using Ni-NTA resin (Qiagen). Elution was performed stepwise with increased imidazole concentration in elution buffer A (50 mM Tris-HCl pH 8, 150 mM NaCl, 100 mM Imidazole, 10 mM β-mercaptoethanol, 10% glycerol, 10 μM ZnCl_2_), elution buffer B (50 mM Tris pH 8, 150 mM NaCl, 150 mM imidazole, 10 mM β-mercaptoethanol, 10% glycerol, 10 μM ZnCl_2_) and elution buffer C (50 mM Tris pH 8, 150 mM NaCl, 250 mM imidazole, 10 mM β-mercaptoethanol, 10% glycerol, 10 μM ZnCl_2_). Fractions containing TRIM21 B-box2 were dialyzed in buffer over-night and the 6xHis-tag was cleaved with thrombin during the dialysis (50 mM Tris-HCl pH 8, 150 mM NaCl, 10 mM β-mercaptoethanol, 10% glycerol and 10 μM ZnCl_2_). Further purification was performed on a Hiload Superdex 75 gel filtration column (GE Healthcare) to remove residual impurities. Protein purity was analyzed by SDS-PAGE. Prior to NMR measurements, protein samples was concentrated using concentrators with a 3000 molecular weight cut-off (Amicon Ultra, Millipore) to a final concentration of 450 μM. Stable protein NMR samples for assignment and structure calculation were prepared in 50 mM Tris-HCl, 150 mM NaCl, 10 mM β-mercaptoethanol, 10 μM ZnCl_2_, 90% H_2_O/10% D_2_O at pH 7.5. Higher Zn^2+^ concentrations resulted in unstable samples not suitable for NMR analysis. The requirement of Zn^2+^ for the protein to fold was evident from the protein production and purification protocol, which had to be carefully optimized with respect to Zn^2+^, pH and reducing agents to avoid aggregation and/or lack of expression. Similar protocol was followed for purifying TRIM21_1-91_ construct comprising the RING domain and its flanking helices [22].

### NMR spectroscopy

NMR spectra were recorded at 25°C on Varian INOVA 600 and Bruker AvanceIII 800 MHz spectrometers. All spectrometers were equipped with a cryogenically cooled probe-heads. Standard pulse sequences for HNCO, HNCA, HN(CO)CA, HN(CA)CB and CBCA(CO)NH were used to record spectra for backbone resonance assignment. Side chain resonances were assigned from the ^13^C-^1^H correlation experiments of aliphatic and aromatic CT-HSQC, (H)CCH-TOCSY and H(C)CH-TOCSY. The ^13^C-^1^H correlation experiments were acquired using non-uniform sampling (NUS) [23,24]. NOE distance constraints were obtained from ^15^N-NOESY-HSQC experiments collected with a mixing time of 100 ms, and aliphatic and aromatic ^13^C-NOESY-HSQC acquired using mixing times of 100 and 120 ms respectively.

Chemical shift perturbations (CSPs) for TRIM21 B-box2 were acquired from ^15^N-HSQCs on adding unlabeled RING_1-91_. ^15^N-HSQC spectra were recorded at 0.25, 0.5, 1.0 and 2.0 equivalents of RING_1-91_. The protein concentration of TRIM21 B-box2 was 200 μM. The difference in ^1^H and ^15^N chemical shifts between the apo TRIM21 B-box2 and the TRIM21 B-box2 bound to RING_1-91_ complex was calculated using the equation [25]:

Δδcomp= [ΔδNH2+ (ΔδN/6.5)2]12 (1)

The relaxation experiments ^15^N-R_1_, ^15^N-R_1ρ_ and {^1^H}-^15^N-NOE were recorded at 600 MHz using standard pulse sequences [26] [27] for the TRIM21 B-box2 using a protein concentration of 150 μM. For the R_1_ experiments, 19 data points were recorded, using relaxation delays ranging between 10 and 646 ms. A total of 18 data points were recorded for the R_1ρ_ experiments, with relaxation delays between 6 and 100 ms. The spin lock field strength was 1761 Hz and the carrier was set to 118.98 ppm during spin lock. The {^1^H}-^15^N-NOE was measured by taking the ratios of peak intensities in experiments including or not including a 5 s period of ^1^H saturation pulses. The total recovery delay was 12 s for both experiments. Using the following equation, R_2_ was calculated from the R_1_ and R_1ρ_ experiments:

$$R_{1\rho }=\;R_{1}{\text{cos}}^{2}\theta +R_{2}{\text{sin}}^{2}\theta$$ (2)

where *θ* = arctan(*B*_*1*_/Ω) is the tilt-angle of the effective field with respect to the static magnetic filed, where *B*_*1*_ is defined as the spinlock field strength in frequency units and Ω is the resonance offset from the radio frequency carrier [28].

All NMR data were processed with NMRpipe [29] and MddNMR [24], which uses multidimensional decomposition and compressed sensing to reconstruct the data. The spectra were visualized with SPARKY (Goddard & Kneller, University of California, San Francisco). The software PINT [30] was used to perform the peak integration, to fit R_1_ and R_1ρ_ and finally to calculate R_2_ and heteronuclear NOE. All structure figures were made using PyMOL (http://www.pymol.org/).

### Structure calculations

Distance restraints for structure calculations were derived from cross-peak NOEs in ^15^N-NOESY-HSQC, aliphatic ^13^C-NOESY-HSQC and aromatic ^13^C-NOESY-HSQC respectively. Peak picking was performed manually using SPARKY. The restraints for the *φ* and *ψ* backbone dihedral angles were predicted empirically from chemical shifts of backbone atoms (NH, H, Cα, Cβ, Hα and Hβ) using the TALOS software [31].

During the structure determination, NOESY peaks were assigned in iterative cycles of automated structure calculations and NOE assignment using the software CYANA 2.1 [32]. The initial structures were calculated without including zinc ion coordination restraints and using only dihedral angle and NOE distance constraints, until the structures were well converged. The Zn^2+^-coordinating residues were identified by analysis of the chemical shift of potential Zn^2+^-coordinating cysteines [33] together with sequence alignments, and the initial structure ensembles that were calculated using only dihedral angle and NOE distance restraints. Once the fold was obtained, Zn^2+^ ions were introduced to the structure using virtual linkers and restraints between pair of atoms (Zn-Sγ, Sγ-Cβ, His Nε2-Sγ and Sγ-Sγ) were enforced to maintain a proper tetrahedral geometry around the zinc atom [34]. The nonexperimental distance restraints between atom pairs were restrained within bounds as previously described [35]. No additional NOE violations were produced after the introduction of the zinc ion constraints. The final ensemble of the 20 lowest-energy structures out of 100 in the final cycle was refined using the CNS package [36] by performing a short constrained molecular dynamics simulation in explicit solvent. The quality of the final structures were analyzed using MOLMOL [37] and the PSVS validation software packages [38], including global quality factor from Verify 3D, ProsaII, PROCHECK [39] and MolProbity [40]. The global goodness-of-fit of the final structure ensemble of the NOESY peak list data was calculated using RPF analysis [41].

### Structural and bioinformatic analysis

The chemical shift index (CSI) of backbone atoms can be used to identify the secondary structural elements of a specific protein [42]. The CSI of Cβ and NH atoms provides a prediction of protein secondary structures, while the CSI of Cα and Hα atoms can be used to identify secondary structural elements as α-helices and β-sheets. Backbone chemical shifts and sequence data was used as input to the CSI 3.0 server in order to identify secondary structure [43]. Random coil shift were predicted using the ncIDP (Neighbor Corrected IDP Library) server [44] based on the amino acid sequence of TRIM21 B-box2_86-130_. The chemical shift values of the NH, H, Hα, CO, Cα and Cβ atoms of the assigned residues were compared to the corresponding predicted random coil values.

The secondary structure server (2Struc) [45] was used to analyze the presence of secondary structure elements in the TRIM21 B-box structure using the Dictionary of Secondary Structure of Proteins (DSSP) algorithm [46].

Conserved residue analysis was performed using ConSurf, which uses evolutionary information to identify functional regions of a protein domain [47,48]. Multiple sequence alignment of available B-box type2 PDB structures was created using MUSCLE (3.8) [49]. A Profile hidden Markov Model (HMM) logo was constructed for TRIM21 B-box_91-130_. The HMM logo was built from the alignment of the zinc finger B-box type region (PROSITE-ProRule annotation PRU00024; Pfam zf-B_box PF00643) of available B-box type2 PDB structures and submitted to the WebLogo server [50] in order to generate the logo. VADAR (Volume Area Dihedral Angle Reporter) [51] was used for structure evaluation including hydrogen bonding partners and accessible surface area. Side chains were considered buried if level of exposure was less than 15%. Structural analysis of interfaces was obtained using the PDBePISA (Proteins, Interfaces, Structures and Assemblies) server [52]. A homology model of TRIM21 B-box2-coiled-coil dimer (residues 86–253 in chain A and B) was constructed by first using HHpred [53] to search for available templates using HMM-HMM comparisons against pdb70_22Oct16 database. The top scoring template was the structure of TRIM5a B-box-coiled-coil (PDB ID: 4TN3) with E = 1.2e-21, the gapless alignment had 45% sequence identify and covered the whole sequence. Modeller v9.16 [54] was then used to build a multi-chain model of the dimer using the top scoring target-template alignment.

The structure coordinates of the final refined ensemble of 20 TRIM21 B-box2 structures have been deposited in the Protein Data Bank (PDB ID: **5JPX**), and, the ^1^H, ^15^N and ^13^C resonance assignment in the Biological Magnetic Resolution Data Bank (BMRB) with accession code **30075**.

## Results

### TRIM21_86-130_ adopts a B-box fold with one Zn^2+^ site occupied

**Fig 1:**
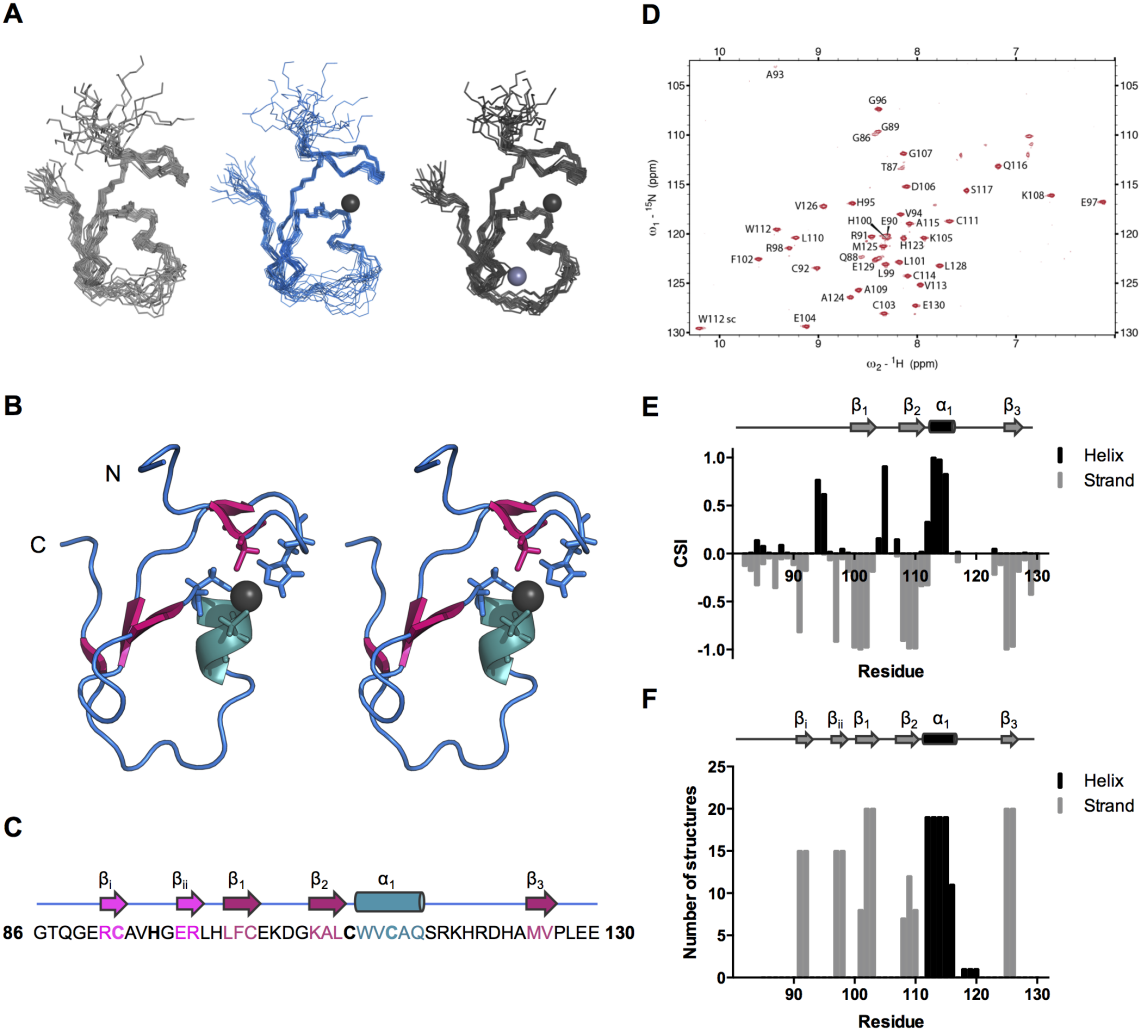
Structural ensemble and secondary structure of TRIM21 B-box2. (A) The backbone trace of the structural ensembles of the 20 best-fit NMR structures of no Zn^2+^ (grey), 1 Zn^2+^ (blue) and 2 Zn^2+^ (dark grey). The ensemble is aligned based on the ordered residues as reported by PSVS (Zn0: residues 91–117, 125–128; Zn1: residues 91–117, 125–128; Zn2: residues 90–118; 122–128). (B) Stereo view of the tertiary structure of TRIM21 Bbox2. Zinc atoms are shown as grey spheres and zinc-coordinating residues in Zn^2+^ site1 are highlighted as sticks. (C) The amino acid sequence of TRIM21 B-box2_86-130_. Zinc-coordinating residues in Zn^2+^ site1 are highlighted in bold face. (D) ^15^N-HSQC spectrum of the TRIM21 B-box2, displaying a well-folded domain. (E) Identification of secondary structure elements by the Chemical Shift Index (CSI) *per* residue. (F) Secondary structure elements assigned by DSSP.

Given the well-resolved NMR spectra of TRIM21_86-130_ comprising the B-box2 motif, we employed NMR to determine its domain structure (Fig 1). Initial structures were determined based on NOE restraints only, in the absence of additional zinc ion restraints in order to not bias the fold of the domain. These initial structures revealed that Cys92, His95, Cys111 and Cys114 were clustered and oriented in a fashion that would enable coordination of Zn^2+^ in site I, as predicted from sequence (Fig 1). In agreement with this, introducing ligand-restraining constraints for site I alone led to improved quality of this ensemble of structures both over that in the absence of Zn^2+^ and where both sites were restrained, further supporting the hypothesis of a single, fully occupied Zn^2+^ site I (Fig 1, Table 1). In agreement, the ^13^Cα and ^13^Cβ chemical shifts of Cys92, Cys111 and Cys114 indicate that these cysteines are coordinating Zn^2+^ with the probabilities of 0.69, 0.49 and 1.00 respectively [33]. Only one Zn^2+^ binding site was observed for the isolated TRIM21 B-box2 in our earlier study [55]. Under the experimentally accessible conditions (10 μM Zn^2+^, pH 7.5), amide resonances of Arg118-Asp122 were not possible to assign due to line broadening, and chemical shift analysis [33] showed zero probability for Cys103 to participate in Zn^2+^ coordination. A second Zn^2+^ site, with proposed ligands Cys103, Asp106, His120 and His123, was therefore deemed to be incompletely saturated, in agreement with a lower expected affinity for a Zn^2+^ site with fewer coordinating cysteines [56]. In agreement, single B-box2 structures determined by NMR show similar chemical shifts for tentative Zn^2+^ ligands in the second site (S1 Fig).

**Table 1:** Structural statistics of the 20 best-fit NMR structures of TRIM21 B-box2 no Zn^2+^, 1 Zn^2+^ and 2 Zn^2+^.

TRIM21 B-box2 NMR structures	No Zn^2+^	1 Zn^2+^	2 Zn^2+^
***A*. *NMR restraints***			
*Distance restraints*			
Total NOE	709	712	686
Intra-residue (i-j = 0)	157	158	150
Sequential (i-j = 1)	221	226	222
Medium-range (1<i-j<5)	115	122	114
Long-range (i-j≥5)	216	206	200
Zinc coordination restraints	0	11	20
*Dihedral angles restraints*			
φ	29	29	29
ψ	29	29	29
***B*. *Structure statistics***			
*Violations*			
Distance (>0.5 Å)	0	0	0
Dihedral angle (> 10°)	0	0	0
*Ramachandran Statistics*^a^			
Most favored regions (%)	97.6	96.1	87.2
Allowed regions (%)	2.4	3.9	12.1
Disallowed regions (%)	0.0	0.0	0.7
*Average pairwise r*.*m*.*s*.*d*. *(Å)*^b^			
Heavy	1.61±0.22	1.31±0.17	1.73±0.24
Backbone	0.80±0.18	0.63±0.14	0.84±0.19
*Global quality score*^c^			
Raw score			
Procheck (phi-psi)^a^	-0.50	-0.60	-0.84
Procheck (all)^a^	-0.37	-0.41	-0.63
MolProbity clash	12.65	10.71	11.65
Z score			
Procheck (phi-psi)^a^	-1.64	-2.05	-2.99
Procheck (all)^a^	-2.19	-2.42	-3.73
Molprobity clash	-0.65	-0.31	-0.47
*RFP scores*^d^			
Recall	0.902	0.902	0.897
Precision	0.889	0.882	0.881
DP-score	0.726	0.715	0.708

^a^ Values calculated for the ordered regions, as reported by PSVS [38] (no Zn^2+^: residues 91–117, 125–128; 1 Zn^2+^: residues 91–117; 125–128; 2 Zn^2+^: residues 90–118; 122–128)

^b^ Calculated for residues 91–128

^c^ Calculated by PSVS [38]

^d^ RPF scores [41] reflecting the goodness-of-fit of the structural ensemble to the NMR-data

The final ensemble of 20 structures displays a well-structured core domain with a ββαβ fold, comprising a central α-helix (α_1_, residue W112-Q116) and a three-stranded β-sheet (β_1_, residue L101-C103; β_2_, residue K108-L110; β_3_, residue M125-V126), in full agreement with chemical shift based CSI evaluation of secondary structure (Fig 1). Furthermore, a β-β-pairing arrangement of two short strands (β_i_, residue R91-C92; β_ii_, residue E97-R98) is noted by DSSP in 15 out of the 20 structures in the ensemble (Fig 1). Chemical shifts and lack of long-range NOEs indicate a flexible N-terminus and in agreement, residues G86-E90 are disordered in the NMR ensemble (Fig 1).

**Fig 2:**
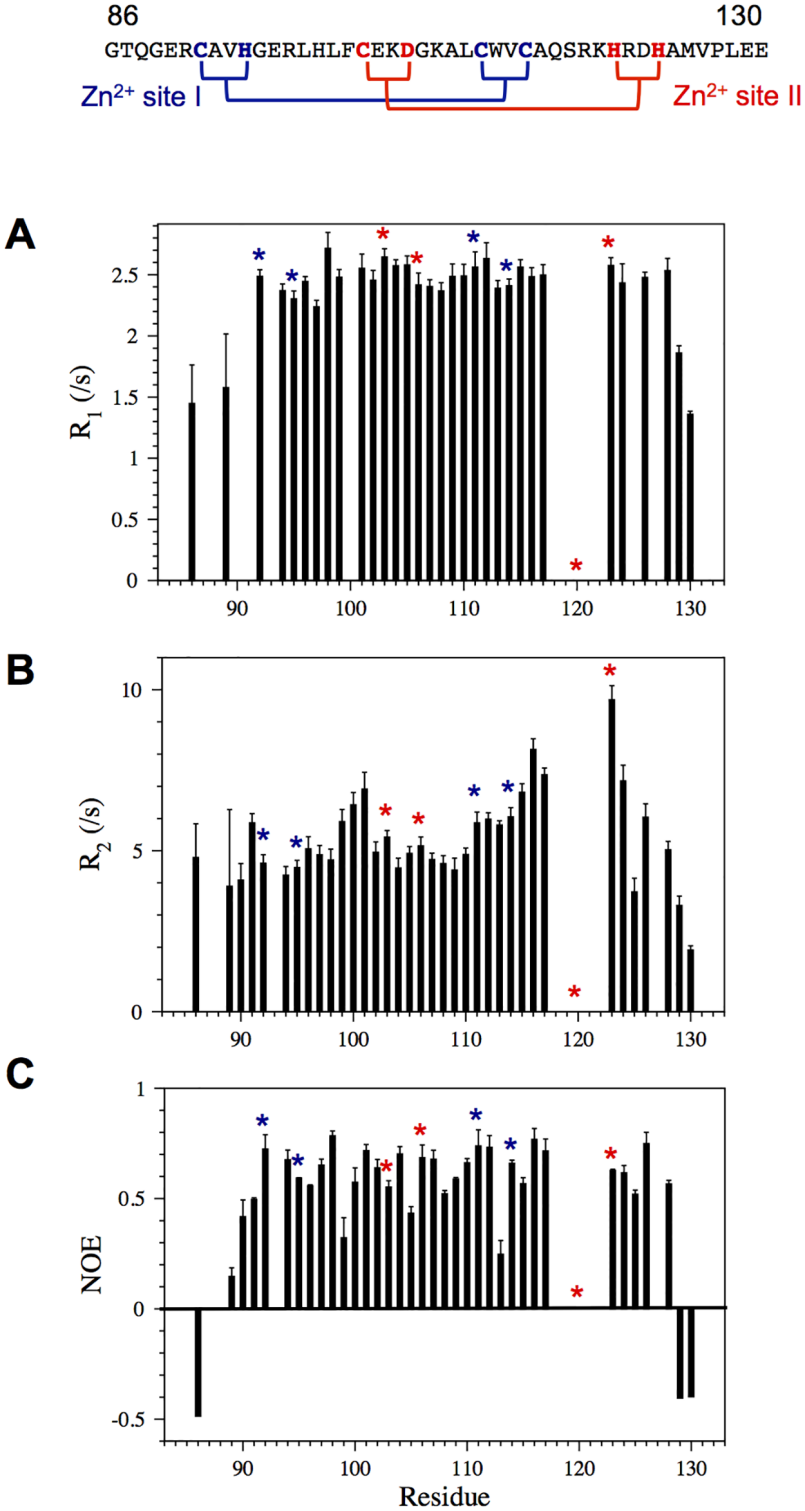
Relaxation data for TRIM21 B-box2. (A) R_1_ relaxation rate constants, (B) R_2_ relaxation rate constants and (C) the heteronuclear NOE. Residues involved in Zn^2+^ coordination are highlighted in blue (site I) and red (site II). In site I, one zinc atom is coordinated by three cysteines and one histidine. In site II, one cysteine, one aspartic acid and two histidines would coordinate a second zinc atom.

To investigate the dynamics of TRIM21 B-box2, ^15^N-R_1_, ^15^N-R_2_, and {^1^H}-^15^N-NOE relaxation experiments were evaluated. Within the core fold, the B-box displays only small fluctuations in both ^15^N-R_1_ and ^15^N-R_2_ rates and the positive {^1^H}-^15^N NOE relaxation data of about 0.6 indicates a well-folded core domain, including all of the coordinating residues of zinc site I, and two of the proposed coordinating residues of Zn2+ site II (Fig 2). Increased R_2_ values for residues flanking the unassigned Arg118-Asp122 region, in particular for His123, support possible μs-ms exchange in this loop (Figs 1A and 2B), possibly affected by intermediate exchange of Zn^2+^ to a lower-affinity site II (Fig 2). From the R_2_/R_1_ ratios of the core domain we estimated the correlation time (τ_c_) for molecular reorientation to 3.4 ns, which agrees with expectations for a monomeric protein of this size at 25°C [57]. This conclusion was corroborated by hydrodynamic calculations [58].

### The TRIM21 B-box structure highlights a conserved hydrophobic core

**Fig 3:**
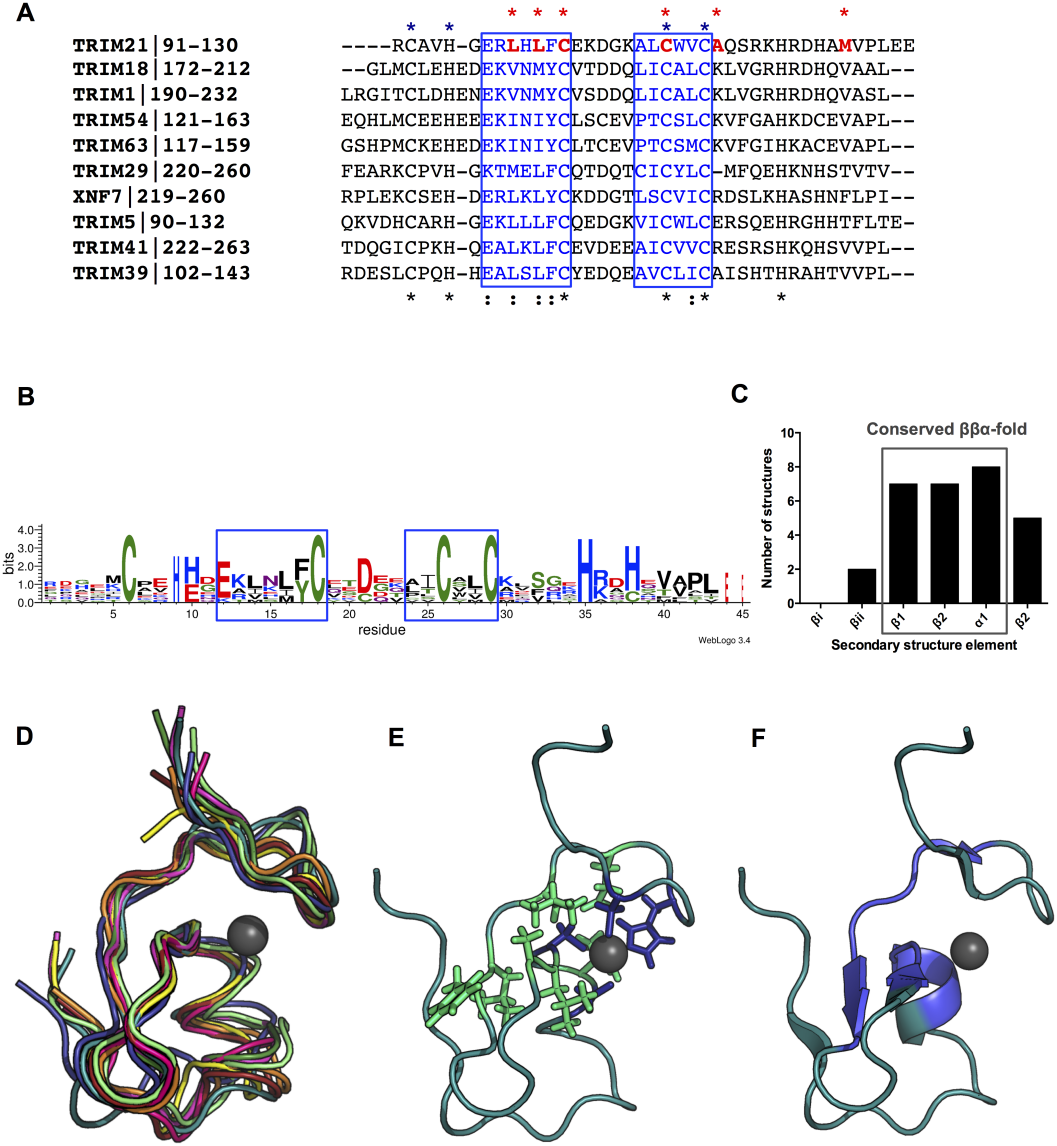
Structural analysis of TRIM family type2 B-boxes. (A) Sequence alignments of TRIM family showing type2 B-boxes region. Residues with a fractional surface accessibility side chain score <0.15 in the TRIM21 B-box2 sequence are marked with a red asterix. The zinc coordinating residues in site I in TRIM21 B-box2 are marked with a dark blue asterix. Conserved core residues are highlighted in blue. (B) HMM logo of the conservation of residues among TRIM type2 B-boxes. (C) Frequency of the conserved ββα core fold among TRIM B-box2 structures. (D) Structure alignment of the core residues of TRIM type2 B-boxes. (E) Highlight of conserved hydrophobic core residues (green) and the zinc coordinating residues in site I (dark blue) on the TRIM21 B-box2 structure. (F) Cartoon representation of TRIM21 B-box2 and the conserved core residues (blue).

To evaluate the fold of the TRIM21 B-box the DALI server [59] was used to search for similar 3D folds of structural homologs. The top DALI match was obtained for TRIM 39 (PDB ID: 2DIF, chain A) with a Z-score of 4.5 and a sequence identity of 46%. Other high-scoring motifs (down to Z-score 3.2) include type-2 B-boxes in TRIM proteins TRIM63/MuRF1 (PDB-ID: 3DDT), TRIM5α (PDB-ID: 2YRG) and TRIM54 (PDB-ID: 3Q1D), as well as the zinc-finger B-box of Transcription intermediary factor 1-beta (PDB-ID: 2YVR). Top matches among deposited structures in the Protein Data Bank database as identified by the PDBsum server [60] included TRIM5α (PDB ID: 2YRG), TRIM39 (PDB ID: 2DID), TRIM41 (PDB ID: 2EGM), TRIM29 (PDB ID: 2CSV), TRIM18/MID1 B-box2 (PDB ID: 2JUN), TRIM1/MID2 B-box2 (PDB ID: 2DJA) and the XNF7 B-box2 (PDB ID: 1FRE). To investigate this further, the solution structure of TRIM21 B-box2 domain was compared with the solution structures of TRIM1/MID2 B-box2 (PDB ID: 2DJA), TRIM5α (PDB ID: 2YRG), TRIM18/MID1 B-box2 (PDB ID: 2JUN), TRIM29 (PDB ID: 2CSV), TRIM39 (PDB ID: 2DID), TRIM41 (PDB ID: 2EGM) and TRIM63/MuRF1 (PDB ID: 3DDT). The core region of all the structures has a similar overall fold when compared to the TRIM21 B-box2 (Fig 3). Most strikingly, the orientation of the secondary structure elements and the location of the Zn^2+^ ions are alike. Most structures display the same ββαβ fold as TRIM21, but TRIM18 and TRIM1 lack the third β-strand (β3) and only show a ββα fold. The orientation and arrangement of Zn^2+^-ligated residues in site I and site II all comprise a cross-braced Zn^2+^ arrangement as in TRIM21 (S1 Fig).

In addition to the Zn^2+^ binding sites, a second determinant for the fold conservation is a pattern of hydrophobic residues that interconnects the secondary structure elements (Fig 3). This interconnected network includes the Zn^2+^-coordination residues for site I, together with a set of buried, conserved residues in β_1_ and β2 and α_1_ as annotated (Fig 3A). The same hydrophobic core is conserved in a set of B-box structures (Fig 3A and 3B). Furthermore, in a Consurf analysis, featuring a multi-sequence alignment of homologous structures from the Uniprot database, the same conservation pattern is evident (S2 Fig). Interestingly, this hydrophobic core is exposed at two main hydrophobic patches on the surface of the protein domain. The first hydrophobic patch comprises residues Leu101, Trp112 and Val113 and is located on the outer surface of the α_1_-helix and the β_1_ strand in the protein core. The residues Trp112 and Val113 constitute the α-helix of the fold. The second hydrophobic patch is located at the outer surface of the anti-parallel β-strands β_2_, β_3_ and β_4_ and is formed by residues Leu99, Phe102, Ala109, Leu110 and Val126. Leu99 and Leu101 fold into the core as evidenced by low accessibility scores (Fig 3A). In addition, the zinc-binding loop of site I display two exposed hydrophobic residues, Ala93 and Val94, located in between the first ligand pair, Cys92 and His95. Similar hydrophobic patches have been observed for TRIM5α [15] and TRIM18/MID1 B-box2 [61], but their relation to an inner conserved core has not previously been noted.

### The TRIM B-box interacts with the RING-RBL region using a conserved surface patch

**Fig 4:**
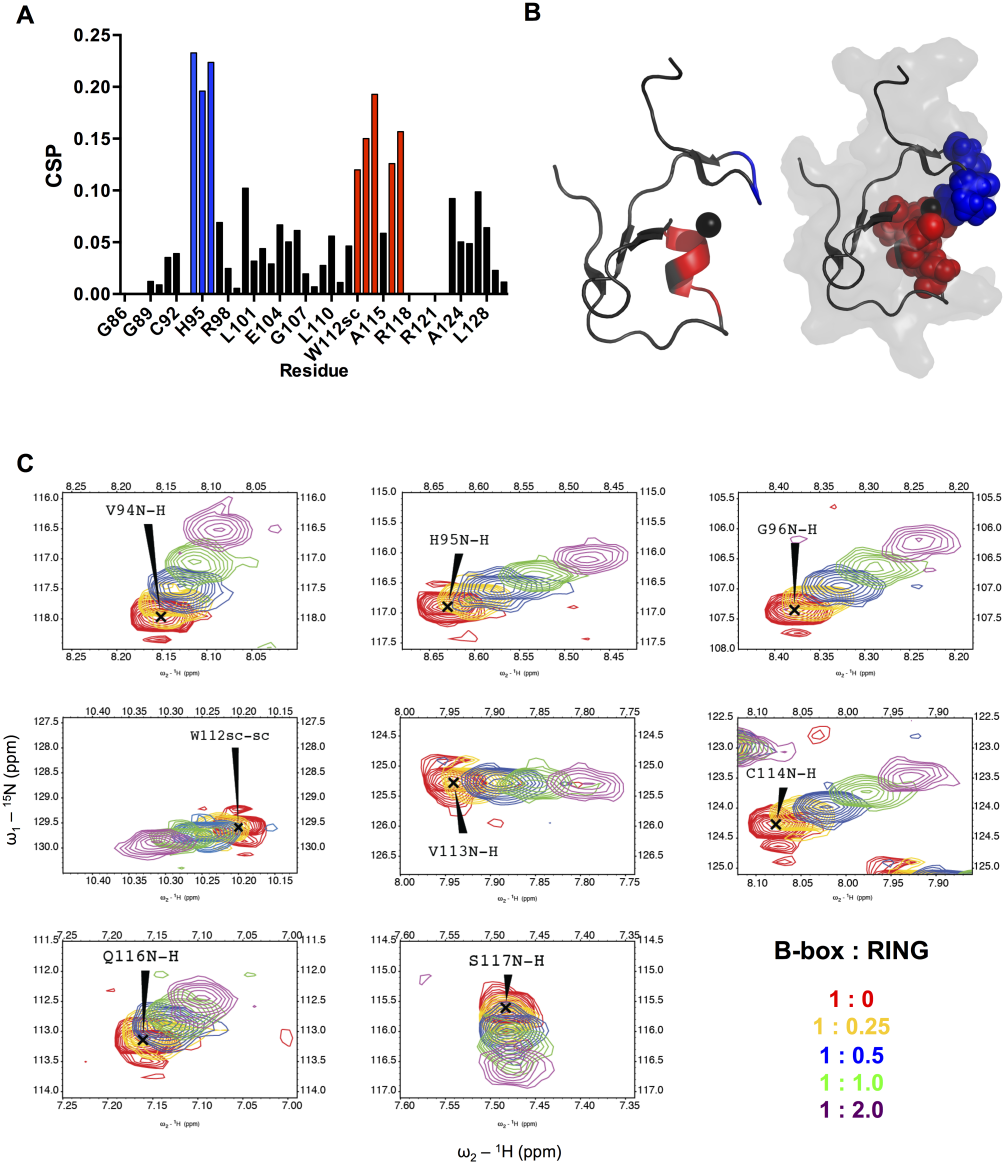
TRIM21 RING_1-91_ titration of TRIM21 B-box2. (A) Normalized chemical shift perturbations (CSP) upon addition of unlabeled TRIM21 RING_1-91_ to ^15^N-labeled TRIM21 B-box2. The CSP cut-off was set to 0.10. Residues are colored as blue (cluster1; V94, H95, G96) and red (cluster2; W112 side-chain, V113, C114, Q116, S117). (B) Mapping of residues on the B-box2 structure affected by the addition of unlabeled TRIM21 RING_1-91_. (C) Highlight of spectral perturbations of N-H shifts of the affected residues V94, H95, G96, W112 side-chain, V113, C114, Q116 and S117.

To investigate the interaction of the TRIM21 B-box2 domain with the N-terminal RING domain on a per-residue level, spectral changes for ^15^N/^13^C-labeled TRIM21 B-box2 upon addition of unlabeled TRIM21 RING_1-91_ were analyzed by ^15^N–HSQC experiments. The observed chemical shift perturbations (CSPs) were mapped on the TRIM21 B-box structure (Fig 4, S3 Fig). Significant CSPs were observed for two clusters of residues. The residues most affected were Val94, His95, Gly96, Val113, Cys114, Gln116 and Ser117. In addition, the side chain amide of Trp112 also displayed a significant shift (Fig 4). Residues Trp112, Val113, Cys114 and Gln116 encompass the α-helical segment of the fold and Ser117 is the first residues in the loop that connects the α-helix (α1) with the third β-strand (β3). All side-chain atoms are facing outwards from the core. Residue Ala115 is also part of the α-helix but is indeed buried and not affected by the addition of RING_1-91_, as supported by its low score of accessible surface area. Residues Val94, His95 and Gly96 together form an exposed stretch and these residues are also situated close to the first Zn^2+^-site. Interestingly, residue His95 is a key residue in the zinc-coordination of this first Zn^2+^-site. Taken together, these two clusters of residues together form a well-exposed patch where the N-terminal RING domain could bind. Thus, these residues constitute an exposed surface intended for protein-protein interaction whilst the surface accessibility of these residues further supports the observations from the CSPs analysis.

### The B-box-RING interaction permits B-box binding to neighboring TRIM domains

**Fig 5:**
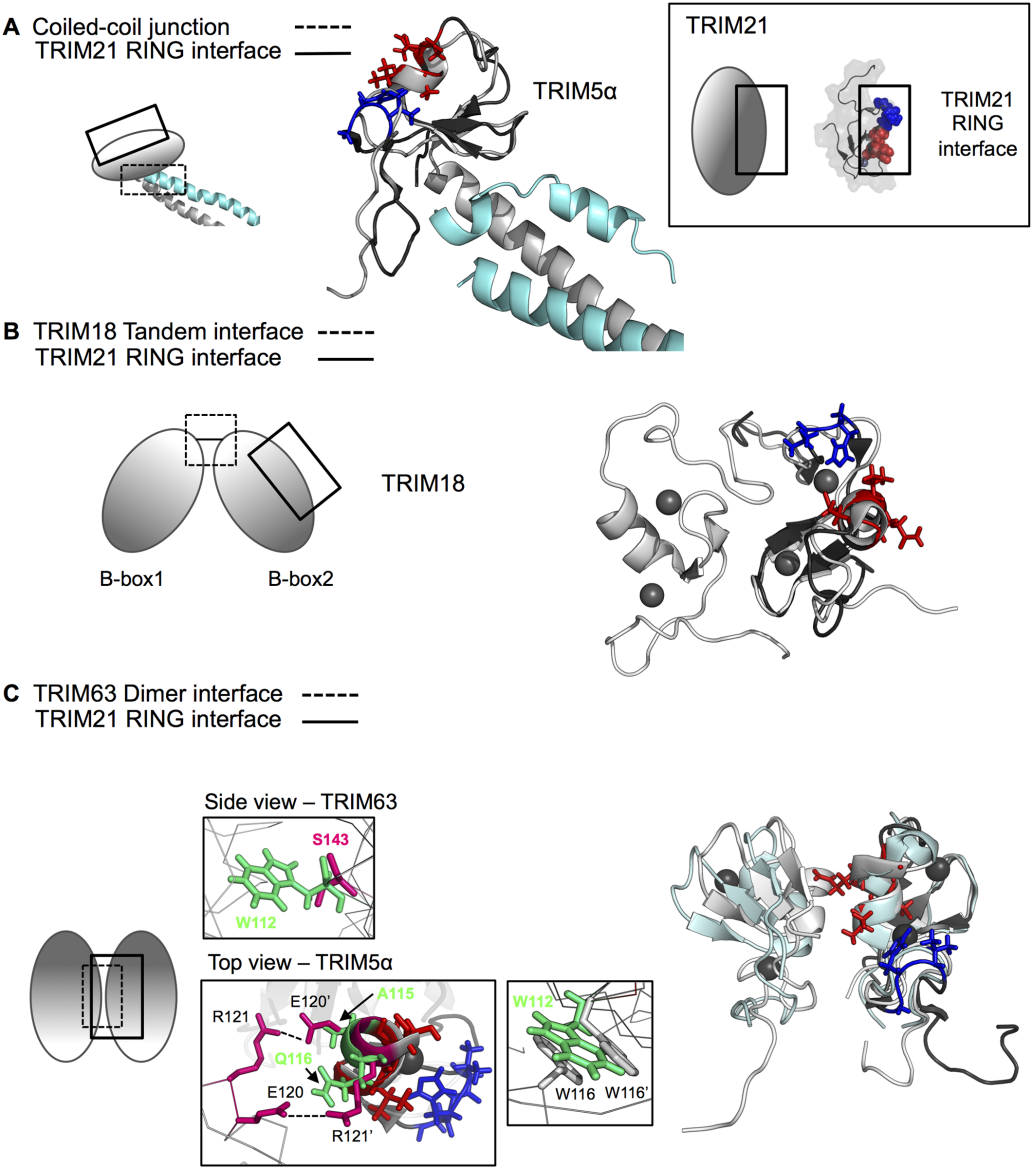
Evidence for a novel RING interaction patch. (A) Superposition of TRIM5α BCC (PDB ID: **4TN3**) chain A (cyan), chain B (grey) and TRIM21 B-box2 (dark grey). Residues in TRIM21 B-box2 affected by TRIM21 RING_1-91_ are highlighted in blue (V94, H95, G96; cluster 1) and red (V113, C114, Q116, S117; cluster 2) in this entire figure. (B) Structural alignment of TRIM18/MID1 tandem Bbox1-Bbox2 (grey; PDB ID: **2DQ5**) and TRIM21 B-box2 (dark grey). Residues involved in TRIM18 Bbox1-2 tandem assembly do not interfere with a tentative RING interaction site as predicted from TRIM21. (C) Structural alignment of TRIM63 B-box2 dimer (ice blue; PDB ID: **3DDT**), TRIM5α B-box2 dimer (grey; PDB ID: **5K3Q**) and TRIM21 B-box2 (dark grey). In TRIM63, S143 in B-box_1_ and B-box_2_ (pink) stack together to form the dimer interface. In TRIM21, there is a tryptophan (green) in this position (W112). The bulky side-chain structure of W112 makes it unlikely that TRIM21 B-box forms a dimer due to steric clashes. In TRIM5α, the dimer is hold together by a salt bridge between R121 and E120. TRIM21 poses an alanine and a glutamine in these positions. The tryptophan W116 is conserved in TRIM21 (W112).

Interestingly, the residues interacting with the TRIM21 RING-dimer motif are located on the opposite side of the TRIM21 B-box2 fold compared to residues that would be predicted to interact with the TRIM21 coiled-coil domain, based on the TRIM5α B-box-coiled-coil structure [13] (Fig 5A). To investigate this further, we made a homologous structure prediction of TRIM21 B-box2-coiled-coil_86-253_ using TRIM5α B-box-coiled-coil dimer (residue 94–258; PDB ID: 4TN3) as template (S4 Fig). The packing of the B-box2 relative to the coiled-coil domain in TRIM5α employs the interaction between residue Phe107 on the B-box2, Val136 and Ala137 on the α2 helix of the first coiled-coil monomer, and Leu249 on α3 of the helical loop 2 region (L2) from the opposing coiled-coil monomer, which creates a hydrophobic interactive core around the B-box β-sheet region. While the sequence of TRIM5α and TRIM21 display the same residues in the β-sheet constituting the hydrophobic core, namely Leu-Phe-Cys, it is likely that the TRIM21 B-box2 shares the same arrangement of B-box-coiled-coil junction as do TRIM5α. Assuming that the observed B-box2 CSPs on RING titration reveals the interaction between B-box and RING domains, the RING-interacting motif of the B-box is then located on the opposite side of the domain (Fig 5A). Furthermore, B-box domains can associate on an intermolecular level. The tandem orientation of B-box1 and B-box2 in TRIM18/MID1 [12] constitute an intermolecular association where the two B-boxes pack against each other, similar to the RING heterodimer of BRCA1-BARD1 [62]. Since the residues of the TRIM18/MID1 B-box2 involved in the association are located on the opposite face of the B-box2 domain relative to the TRIM21 B-box2 residues affected by the addition of RING_1-91_ (Fig 5B), the RING interaction pattern described here could also make sense for TRIM18/MID1.

### B-box TRIM63/5α homodimers employ the TRIM21 B-box RING interface for interaction

While we find that the relaxation properties of the TRIM21 B-box2 agrees with a monomer in solution, two B-box2 dimer structures have been presented [16]; [63] in line with the hypothetical role of the B-box in oligomerization. The TRIM63 B-box2 crystal structure shows a dimer with a highly conserved dimerization interface, where hydrophobic interactions are formed between α1 helix of the first subunit and β1 and β3 sheets of the second subunit, and polar contacts are observed for residues E128-N131 and by the close packing of residue S143 from the α1-helix of both subunits [16]. However, the TRIM21 B-box2 has a distinctly conserved tryptophan in the same position as the highly conserved Ser143 of TRIM63, which would directly obscure the reciprocal close packing of the α1-helices that is a key feature of the TRIM63 B-box2 dimer interface (Figs 5C and 3A). The crystal structure of the TRIM5α B-box2 domain dimer relies on the formation of a salt bridge between Glu120 on one Bbox-2 monomer and Arg121 on the opposing monomer, supported by a stabilizing Thr130 hydrogen bond [63]. However, the TRIM21 B-box2 lacks the negatively charged Glu120 and the positively charged Arg121 that are involved in salt bridge formation, and are functionally required for oligomerization [15]. Instead, TRIM21 B-box2 poses an alanine (Ala115) and a glutamine (Gln116) at these positions, thus precluding similar hydrogen bonds from being formed. The tryptophan in the dimer interface of TRIM5α is conserved in the TRIM21 B-box2 but likely the orientation of the Trp112 in TRIM21 does not satisfy close packing with the opposing tryptophan in a similar dimer arrangement as in TRIM5α (Fig 5C). To conclude, comparison with internal B-box interaction surfaces displayed by TRIM18/MID1, TRIM5α and TRIM63 reveals that the B-box-RING interaction surface identified for TRIM21 allows for RING binding of a sequentially arranged B-box domain pair of TRIM18/MID1 but would interfere with the interdomain B-box association pattern shown for dimeric B-box2 domains of TRIM5α and TRIM63 (Fig 5B and 5C).

## Discussion

In this work, we have used NMR spectroscopy to determine the solution structure of the monomeric TRIM21 B-box2. The domain adopts a ββαβ protein fold, with a classical topology among the Zn^2+^-finger family of B-box proteins. While the B-box topology is classically characterized by dual cross-braced Zn^2+^ coordination, only one site appears fully bound in the isolated TRIM21 B-box2 domain. This agrees with our previous analysis, which suggests a single binding site in the isolated TRIM21 B-box2, whereas increased Zn^2+^ stoichiometry and affinity in the presence of the TRIM21 RING suggest modulation of ion binding by neighboring domains [55]. The pM to nM range of free Zn^2+^ concentrations in the cytosol with signaling transients of higher zinc ion concentrations [64] suggests that a Zn^2+^-binding response that is modulated by neighboring domains could also respond to signaling transients by modular reassembly.

By NMR titration experiments, we have identified an exposed interaction patch where the B-box2 interacts with the TRIM21 N-terminal RING domain. Using TRIM5α-based modeling for structure prediction of the TRIM21 B-box2-coiled-coil intramodular arrangement, we conclude that the TRIM21 B-box2 can simultaneously interact with the RING on one side of the domain, and with the coiled-coil domain on the other side (Fig 5A). In TRIM proteins with more than one B-box, the identified RING interaction motif is also consistent with a sequential B-box1-B-box2 arrangement (Fig 5B).

Interestingly, the B-box-RING interaction surface that we identify for TRIM21 also overlaps with the B-box interaction surface employed to form inter-oligomeric interactions in other TRIM proteins (Fig 5; [16,63,65]). Many TRIM proteins appear to use similar hydrophobic surfaces of the B-box domain to govern both protein-protein interactions and self-association [15,16,61,63]. Recent structural work has further elucidated the assembly of TRIM5α by engineering of shorter TRIM constructs where the B-box is responsible for trimer interactions [65,66]. In these studies, the B-box domains form a three-fold symmetric vertex and are thereby thought to mediate the trimerization interactions. Multiple TRIM5α proteins can be linked into a hexagonal net [67], where the assembly is mediated by the B-box domain [65,66]. The same B-box surface has however been shown to govern dimeric TRIM5α interactions which has led to the suggestion that the B-box domain would hold a plastic oligomerization interface that can assemble into different oligomeric states [63]. However, our detailed sequence-structure analysis suggests that the TRIM21 sequence itself does not support B-box-directed multimer assembly in the shape that has been structurally described for other TRIMs. While mutations of TRIM5α residues such as E120D and R121E/K blocks self-assembly and thereby decrease the ability of TRIM5α to bind HIV-1 capsid-like complexes [15], corresponding residues are not conserved in TRIM21 (Fig 5C). Together with our observation of the TRIM21 B-box2 as a monomer, it is therefore questionable whether the B-box is instrumental in orchestrating trimeric and/or hexameric arrays as observed for TRIM5α. Alternatively, it is possible that in the presence of other TRIM domains, such as the coiled-coil or the RING, the TRIM21 B-box2 might behave differently and then act as an assembly coordinator. The spatial relation between B-boxes and RING domains is so far unknown, and there are no structures describing a RING-Bbox motif. Possibly, the RING-Bbox bimodular entity in itself encompasses a range of various different interdomain orientations, where plastic interaction surfaces may themselves be competing for different intra-or interdomain binding schemes depending on cellular responses affecting the activities of other domains in the multimodular arrangements of which TRIM proteins take part.

The uniqueness of the B-box in TRIM proteins has raised the question as to its importance as a functional motif. The presence of various, distinct interaction motifs within such a small, highly conserved domain fold as the B-box is unexpected but may provide an evolutionary advantage. Indeed, it is conceivable that the stringently conserved arrangement of Zn^2+^ coordinating residues that stabilize the protein core (Fig 3) allows for a larger evolutionary diversity on the surface of the B-box fold. This would permit the development of alternate and even overlapping interaction surfaces that could be utilized both within the modular proteins where the B-box is located, and between proteins with which it interacts. Utilizing this possibility for evolutionary variation, provided by the stability- and fold-conserving Zn^2+^ binding fold, the B-box would then act as a versatile interdomain joint that could support various intermodular arrangements. Such versatility may provide a clue to the evolutionary success of the TRIM protein family and their participation in a wide array of biomolecular functionalities.

The identity of the B-box2 residues proposed to interact with the RING-dimer in our study suggests that the B-box2 despite its small size is a versatile component that may be involved in defining the quaternary modular arrangement both within and between TRIM proteins. Given its modular position between RING and coil-coiled domains, it is possible that the B-box2 has a functional role in regulating RING-mediated ubiquitination as well as interactions with other proteins. The current work brings to attention the possible dual functions caused by overlapping interaction surfaces, and how these could contribute to interdomain plasticity. The functional role of such plasticity remains to be investigated.

## Supporting information

S1 Fig Comparison of chemical shifts of Zn^2+^-ligated residues. (A) Correlation between Cα and Cβ chemical shift (in ppm) for residues involved in Zn^2+^ site I and site II for the NMR structures TRIM21 B-box2 (PDB ID: **5JPX**), TRIM18 B-box2 (PDB ID: **2DQ5**) and TRIM1 B-box2 (PDB ID: **2DJA**). Residues marked with an asterix (*) are not assigned in TRIM21 B-box2. (B) Zinc-coordination topology within TRIM21 B-box2. One zinc atom is coordinated by three cysteines and one histidine (site I). One cysteine, one aspartic acid and two histidines coordinate a second zinc atom (site II).
(TIF)

S2 Fig ConSurf analysis of TRIM21 B-box2. (A) Conservation of TRIM21 B-box2. (B) Highlight of highly conserved residues, shown as spheres. (C) Cartoon representation. (D) Highlight of zinc-coordinating residues, shown as sticks. The residue coloring reflects the degree of conservation of the particular residue, raging from dark red (highly conserved) to cyan (variable). Zn^2+^ ions are shown as yellow spheres.
(TIFF)

S3 Fig Full ^15^N-HSQC spectral overlay of TRIM21 RING-to-Bbox titration. (A) Significant chemical shift perturbations of ^15^N-labeled TRIM21 B-box N-H shifts are observed for residues V94, H95, G96, W112 side-chain, V113, C114, Q116 and S117 as described in detail in Fig 4, with 0, 0.25, 0.5, 1.0 and 2.0 equivalents of unlabelled TRIM RING_1-91_. CSPs are also observed for a residue labelled “A”, which holds Cα and Cβ shifts possibly corresponding to the single unassigned histidine H120, but lacks sequential peaks to confirm such an assignment. (B) H120 is located at the C-terminus of the helix perturbed by RING binding. Coloring of perturbed residue clusters 1 (blue) and 2 (red) as in Fig 4.
(TIF)

S4 Fig Homology modeling of TRIM5α_94–258_ and TRIM21_86-253_. (A) The Bbox2-Coiled-coil (BCC) TRIM21 dimer. The B-box2 is highlighted in pink and green for each monomer respectively. The coiled coil domain is highlighted in blue. (B) Structure alignment of the BCC region of TRIM5α_94–258_ (PDB ID: **4TN3**; grey) and TRIM21_86-253_ (pink/green/blue). (C) Close-up view of the B-box-coiled-coil junction showing interacting junction residues of TRIM5a (according to reference [13]). V136 and A137 of coiled-coil strand α2’ and L249 of coiled-coil strand α3 pack against F107 of the B-box to form a hydrophobic core. (D) Conserved residues (F102, A131, A132 and L244) of the tenative junction interface of TRIM21 B-box2-coiled-coil. (E) Sequence alignment of the BCC region of TRIM21_86-253_ and TRIM5α_94–258_. The conserved residues in the B-box-coiled-coil junction are marked with a red asterix (*).
(TIF)

S1 Table Summary of deposited B-box structures in the Protein Data Bank (PDB). B-box sequences entities classified with *Pfam Accession Number PF00642 B-box zinc finger*. Out of 19 entities, only 10 3D structures have a primary citation.
(PDF)
