# Supplementary figures and images for: Solution NMR structure of the TRIM21 B-box2 and identification of residues involved in its interaction with the RING domain
Source: PLoS One. 2017 Jul 28;12(7):e0181551. doi: 10.1371/journal.pone.0181551 (PMC5533445; doi:10.1371/journal.pone.0181551)

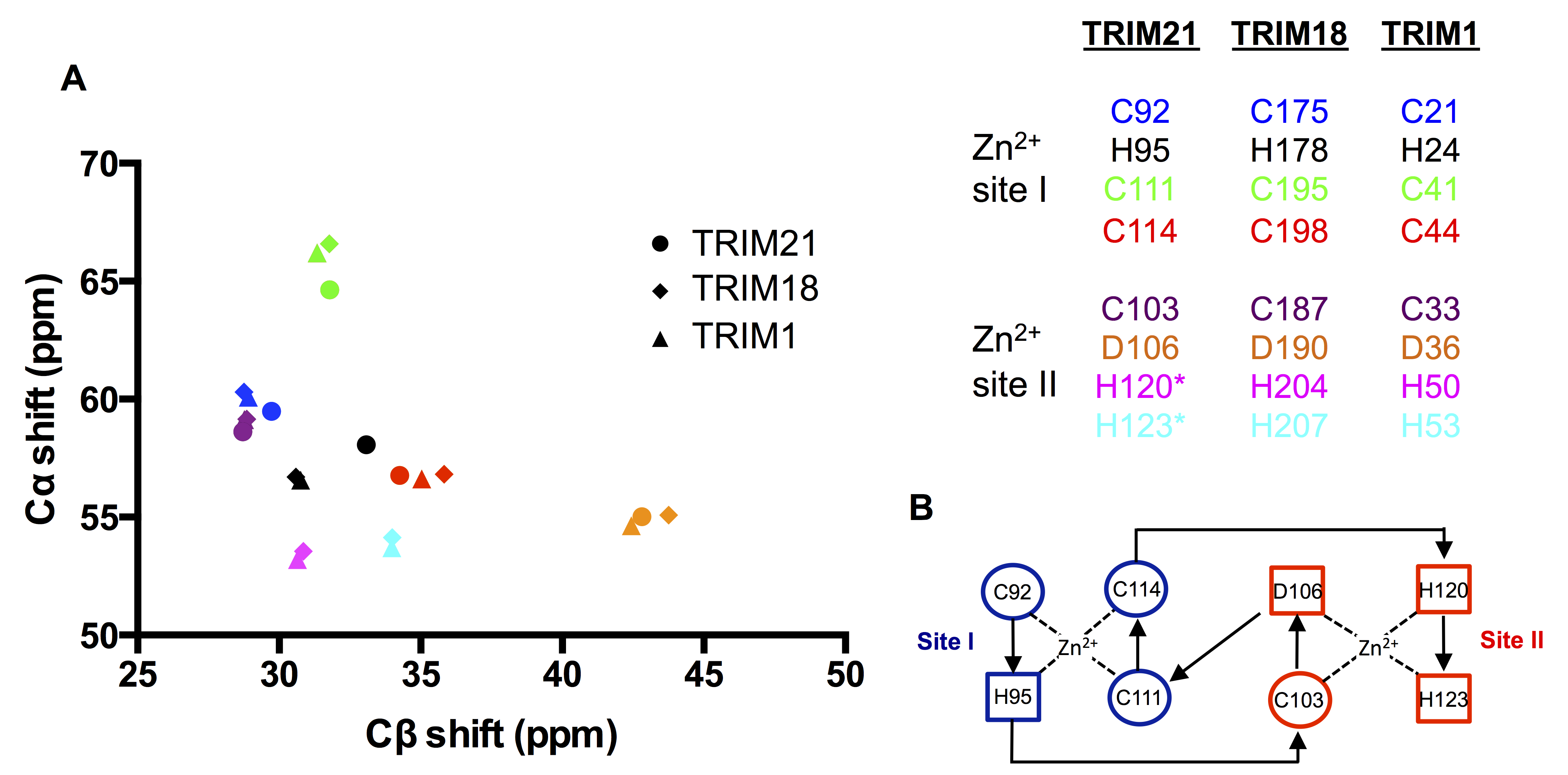

Supplement: S1 Fig — (A) Correlation between Cα and Cβ chemical shift (in ppm) for residues involved in Zn2+ site I and site II for the NMR structures TRIM21 B-box2 (PDB ID: 5JPX), TRIM18 B-box2 (PDB ID: 2DQ5) and TRIM1 B-box2 (PDB ID: 2DJA). Residues marked with an asterix (*) are not assigned in TRIM21 B-box2. (B) Zinc-coordination topology within TRIM21 B-box2. One zinc atom is coordinated by three cysteines and one histidine (site I). One cysteine, one aspartic acid and two histidines coordinate a second zinc atom (site II). (TIF) [file pone.0181551.s001.tif]

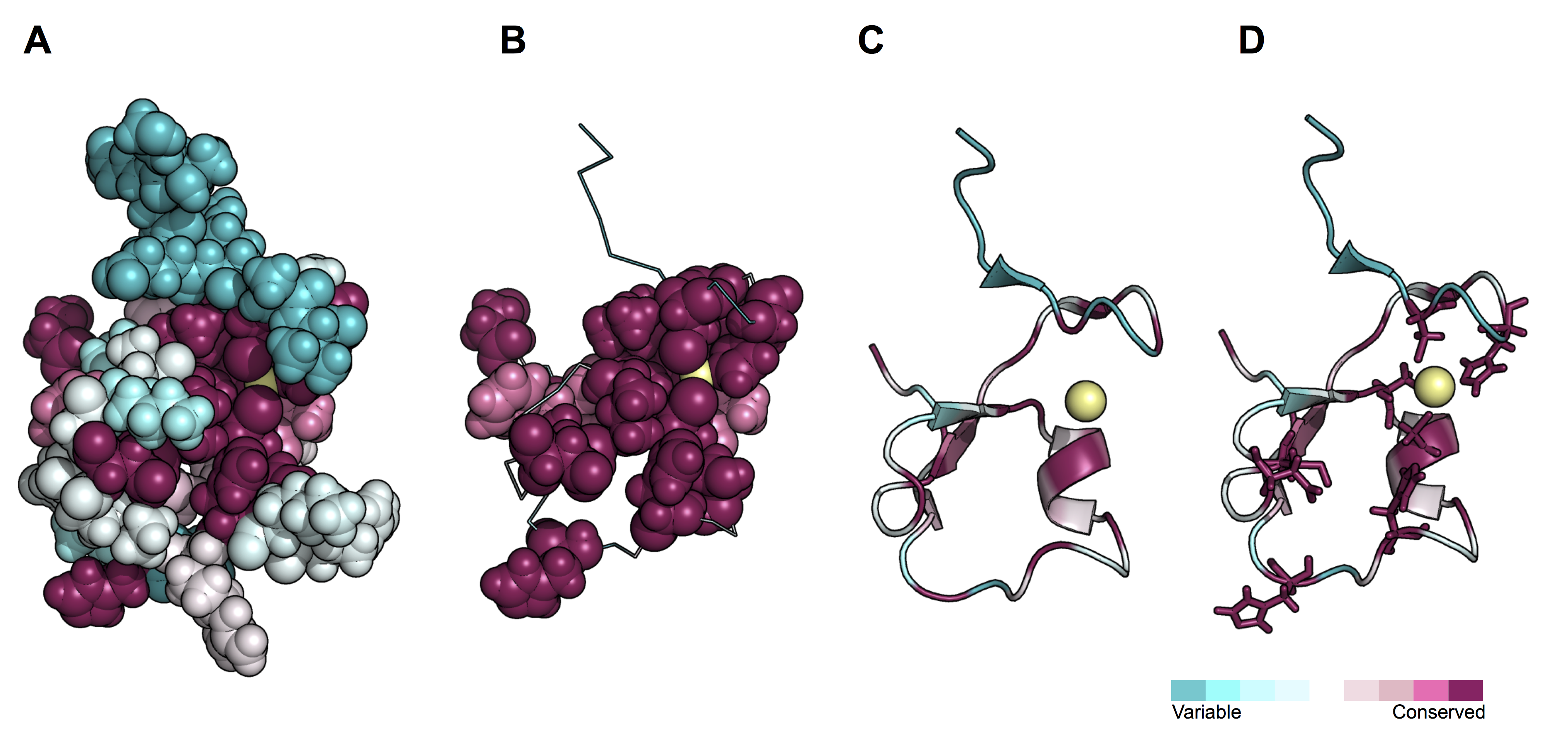

Supplement: S2 Fig — (A) Conservation of TRIM21 B-box2. (B) Highlight of highly conserved residues, shown as spheres. (C) Cartoon representation. (D) Highlight of zinc-coordinating residues, shown as sticks. The residue coloring reflects the degree of conservation of the particular residue, raging from dark red (highly conserved) to cyan (variable). Zn2+ ions are shown as yellow spheres. (TIFF) [file pone.0181551.s002.tiff]

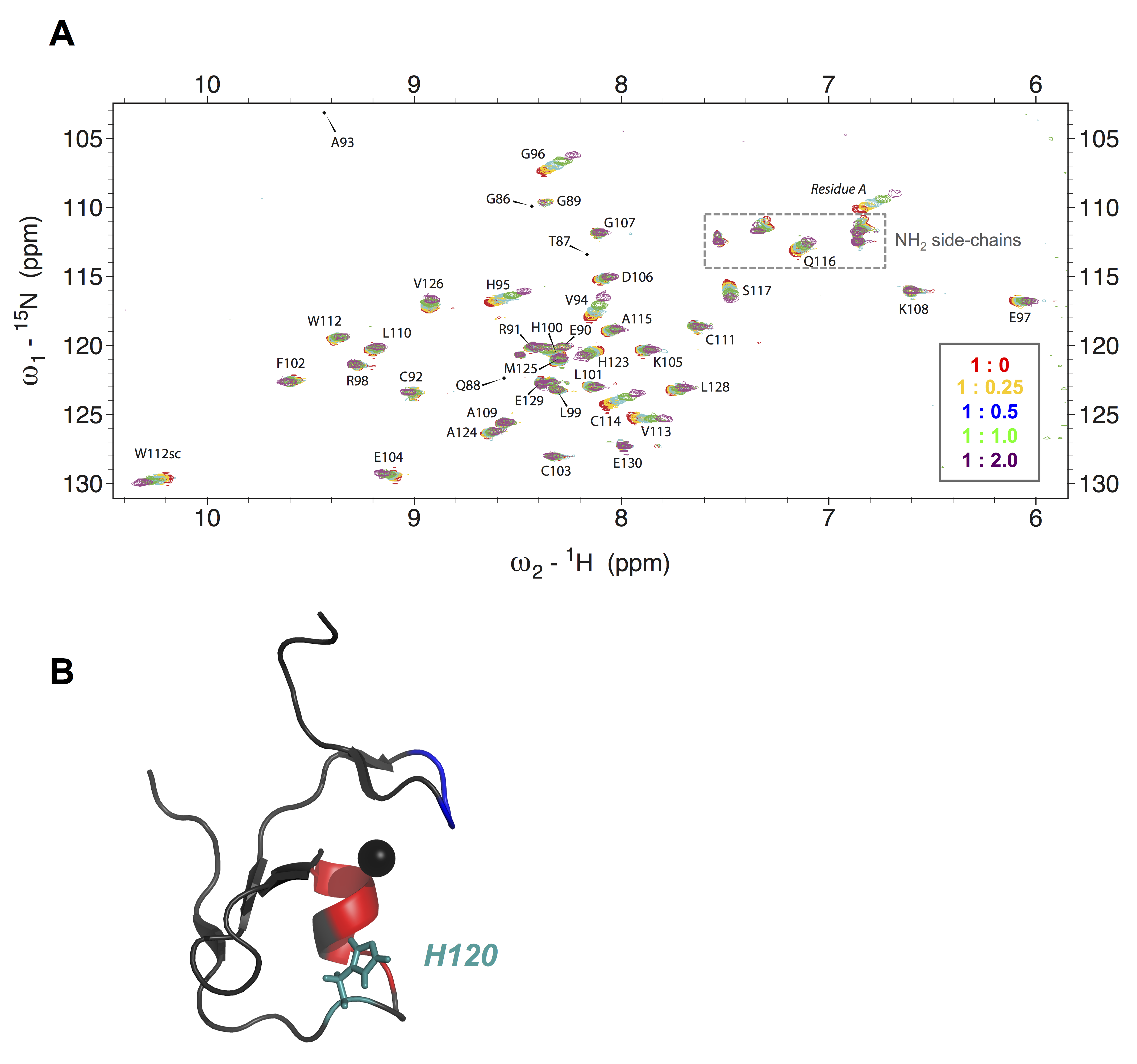

Supplement: S3 Fig — (A) Significant chemical shift perturbations of 15N-labeled TRIM21 B-box N-H shifts are observed for residues V94, H95, G96, W112 side-chain, V113, C114, Q116 and S117 as described in detail in Fig 4, with 0, 0.25, 0.5, 1.0 and 2.0 equivalents of unlabelled TRIM RING1-91. CSPs are also observed for a residue labelled “A”, which holds Cα and Cβ shifts possibly corresponding to the single unassigned histidine H120, but lacks sequential peaks to confirm such an assignment. (B) H120 is located at the C-terminus of the helix perturbed by RING binding. Coloring of perturbed residue clusters 1 (blue) and 2 (red) as in Fig 4. (TIF) [file pone.0181551.s003.tif]

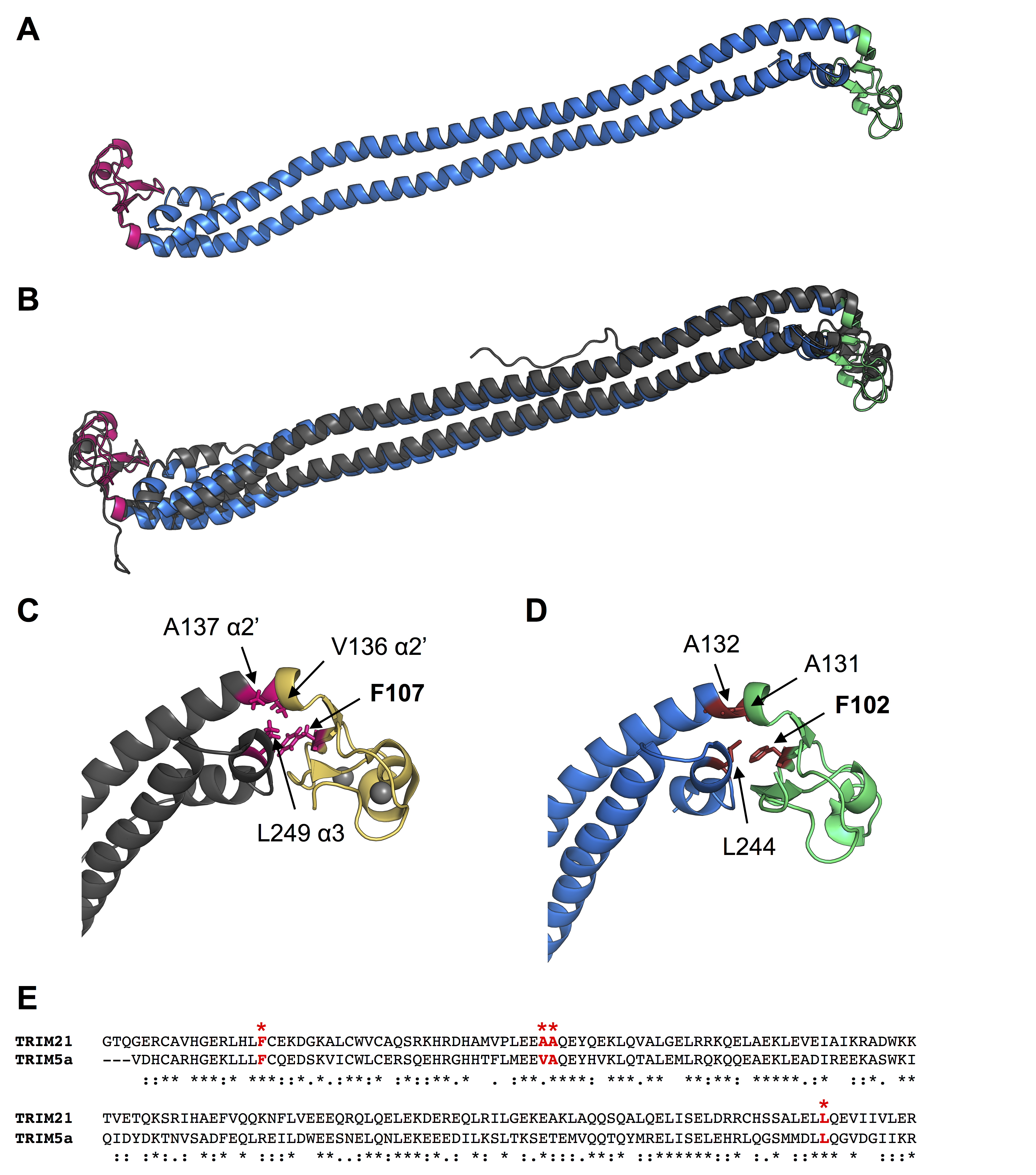

Supplement: S4 Fig — (A) The Bbox2-Coiled-coil (BCC) TRIM21 dimer. The B-box2 is highlighted in pink and green for each monomer respectively. The coiled coil domain is highlighted in blue. (B) Structure alignment of the BCC region of TRIM5α94–258 (PDB ID: 4TN3; grey) and TRIM2186-253 (pink/green/blue). (C) Close-up view of the B-box-coiled-coil junction showing interacting junction residues of TRIM5a (according to reference [13]). V136 and A137 of coiled-coil strand α2’ and L249 of coiled-coil strand α3 pack against F107 of the B-box to form a hydrophobic core. (D) Conserved residues (F102, A131, A132 and L244) of the tenative junction interface of TRIM21 B-box2-coiled-coil. (E) Sequence alignment of the BCC region of TRIM2186-253 and TRIM5α94–258. The conserved residues in the B-box-coiled-coil junction are marked with a red asterix (*). (TIF) [file pone.0181551.s004.tif]
