# Supplementary material for: Solution NMR structure of the TRIM21 B-box2 and identification of residues involved in its interaction with the RING domain
Source: PLoS One. 2017 Jul 28;12(7):e0181551. doi: 10.1371/journal.pone.0181551 (PMC5533445; doi:10.1371/journal.pone.0181551)
Supplement: S1 Table — B-box sequences entities classified with Pfam Accession Number PF00642 B-box zinc finger. Out of 19 entities, only 10 3D structures have a primary citation. (PDF) [file pone.0181551.s005.pdf]

Supplementary Table 1. Summary of deposited B-box structures in the Protein Data Bank (PDB). B-box sequence entities classified with *Pfam Accession Number PF00642 B-box zinc finger*. Out of 19 entities, only 10 3D structures have a primary citation.

Molecule	PDB-ID	Length	Classification	Method	Comment	Primary citation
TRIM5 alpha B-box2	5K3Q	101	Ligase	X-ray diffraction	Dimer (chain A, B)	Keown & Goldstone 2016
Mini TRIM5 B-box2 dimer C2 crystal form	5EIU	141	Ligase	X-ray diffraction	Complex (chain A, D)	Wagner *et al.* 2016
TRIM5 B-box2 and coiled-coil chimera	5F7T	141	Ligase	X-ray diffraction	Complex (chain E, F, H, L)	Wagner *et al.* 2016
TRIM5 B-box2 and coiled-coil chimera	5IEA	141	Ligase	X-ray diffraction	Complex (chain A, B, C, D)	Wagner *et al.* 2016
TRIM19 B-box1	2MVW	51	Metal binding protein	Solution NMR	Dimer (chain A, B)	Huang *et al.* 2014
B-box-Coiled-coil region of Rhesus TRIM5 alpha	4TN3	400	Antiviral protein	X-ray diffraction	Complex (chain A, B)	Goldstone *et al.* 2014
TRIM54 B-box	3Q1D	47	Ligase	X-ray diffraction	Dimer (chain A)	*
TRIM63 B-box2	3DDT	48	Ligase	X-ray diffraction	Dimer (chain A, B, C)	Mrosek *et al.* 2008
Transcription intermediary factor 1-beta	2YVR	50	Metal binding protein	X-ray diffraction	Dimer (chain A, B)	*
MID-1 B-box1 B-box2 tandem	2JUN	101	Ligase	Solution NMR	Monomer (chain A)	Tao *et al.* 2008
TRIM5 B-box2	2YRG	59	Ligase	Solution NMR	Monomer (chain A)	*
TRIM41 B-box	2EGM	57	Transcription /Metal binding protein	Solution NMR	Monomer (chain A)	*
MID1 B-box2	2DQ5	47	Ligase	Solution NMR	Monomer (chain A)	Massiah *et al.* 2007
MID2 B-box	2DJA	84	Metal binding protein	Solution NMR	Monomer (chain A)	*
TRIM39 B-box	2DID	53	Protein binding	Solution NMR	Monomer (chain A)	*
TRIM39 B-box	2DIF	53	Protein binding	Solution NMR	Monomer (chain A)	*
TRIM63 B-box	2D8U	64	Ligase	Solution NMR	Monomer (chain A)	*
TRIM29 B-box2	2CSV	72	Signaling protein	Solution NMR	Monomer (chain A)	*
XNF7 B-box	1FRE	42	Zinc-binding protein	Solution NMR	Monomer (chain A)	Borden *et al.* 1995

* No PubMed ID is available.

References

Borden, K. L., Lally, J. M., Martin, S. R., O'Reilly, N. J., Etkin, L. D., & Freemont, P. S. (1995). Novel topology of a zinc-binding domain from a protein involved in regulating early Xenopus development. The EMBO Journal, 14(23), 5947–5956.

Goldstone DC, Walker PA, Calder LJ, Coombs PJ, Kirkpatrick J, Ball NJ, et al. Structural studies of postentry restriction factors reveal antiparallel dimers that enable avid binding to the HIV-1 capsid lattice. Proc Natl Acad Sci USA. 2014;111: 9609–9614. doi:10.1073/pnas.1402448111

Huang, S.-Y., Naik, M. T., Chang, C.-F., Fang, P.-J., Wang, Y.-H., Shih, H.-M., & Huang, T.-H. (2014). The B-box 1 dimer of human promyelocytic leukemia protein. Journal of Biomolecular NMR. http://doi.org/10.1007/s10858-014-9869-4

Keown JR, Goldstone DC. Crystal structure of the Trim5α Bbox2 domain from rhesus macaques describes a plastic oligomerisation interface. J Struct Biol. 2016;195: 282–285. doi:10.1016/j.jsb.2016.07.004

Massiah MA, Matts JAB, Short KM, Simmons BN, Singireddy S, Yi Z, et al. Solution structure of the MID1 B-box2 CHC(D/C)C(2)H(2) zinc-binding domain: insights into an evolutionarily conserved RING fold. J Mol Biol. 2007;369: 1–10. doi:10.1016/j.jmb.2007.03.017

Mrosek M, Meier S, Ucurum-Fotiadis Z, Castelmur von E, Hedbom E, Lustig A, et al. Structural analysis of B-Box 2 from MuRF1: identification of a novel self-association pattern in a RING-like fold. Biochemistry. 2008;47: 10722–10730. doi:10.1021/bi800733z

Tao H, Simmons BN, Singireddy S, Jakkidi M, Short KM, Cox TC, et al. Structure of the MID1 tandem B-boxes reveals an interaction reminiscent of intermolecular ring heterodimers. Biochemistry. 2008;47: 2450–2457. doi:10.1021/bi7018496

Wagner JM, Roganowicz MD, Skorupka K, Alam SL, Christensen D, Doss G, et al. Mechanism of B-box 2 domain-mediated higher-order assembly of the retroviral restriction factor TRIM5α. Elife. 2016;5. doi:10.7554/eLife.16309
